# Marine algal (seaweed) flora of Faial Island, Azores

**DOI:** 10.3897/BDJ.12.e119875

**Published:** 2024-07-11

**Authors:** David Milla-Figueras, Ana Isabel Neto, Fernando Tempera, Ignacio Moreu, Afonso C. L. Prestes, Roberto Resendes, Raul M.A. Neto, Ian Tittley, Pedro Afonso

**Affiliations:** 1 Okeanos / Institute of Marine Sciences; University of the Azores, Horta, Portugal Okeanos / Institute of Marine Sciences; University of the Azores Horta Portugal; 2 IMAR-Institute of Marine Research, University of the Azores, Horta, Portugal IMAR-Institute of Marine Research, University of the Azores Horta Portugal; 3 Universidade dos Açores, Faculdade de Ciências e Tecnologia, Departamento de Biologia and Grupo de Investigação Aquática Insular, IBBC-GBA/cE3c, Ponta Delgada, Portugal Universidade dos Açores, Faculdade de Ciências e Tecnologia, Departamento de Biologia and Grupo de Investigação Aquática Insular, IBBC-GBA/cE3c Ponta Delgada Portugal; 4 Universidad de Málaga. Instituto andaluz de Biotecnología y Desarrollo Azul (IBYDA). Centro Experimental Grice Hutchinson. Lomas de San Julián, 2 29004, Málaga, Spain Universidad de Málaga. Instituto andaluz de Biotecnología y Desarrollo Azul (IBYDA). Centro Experimental Grice Hutchinson. Lomas de San Julián, 2 29004 Málaga Spain; 5 Universidade dos Açores, Faculdade de Ciências e Tecnologia, Departamento de Biologia and Grupo de Investigação Aquática Insular, IBBC-GBA/cE3c, Ponta Delgada, Açores, Portugal Universidade dos Açores, Faculdade de Ciências e Tecnologia, Departamento de Biologia and Grupo de Investigação Aquática Insular, IBBC-GBA/cE3c Ponta Delgada, Açores Portugal; 6 Universidade dos Açores, Faculdade de Ciências e Tecnologia, Departamento de Biologia, Rua da Mãe de Deus, 13-A, 9500-321, Ponta Delgada, Portugal Universidade dos Açores, Faculdade de Ciências e Tecnologia, Departamento de Biologia, Rua da Mãe de Deus, 13-A, 9500-321 Ponta Delgada Portugal; 7 n/a, Ponta Delgada, Portugal n/a Ponta Delgada Portugal; 8 Natural History Museum, Cromwell Road, SW7 5BD, London, United Kingdom Natural History Museum, Cromwell Road, SW7 5BD London United Kingdom

**Keywords:** macroalgae, taxonomical records, new records, georeferenced occurrence data

## Abstract

**Background:**

A comprehensive checklist of the marine flora from Faial Island, mid-north Atlantic, is still unavailable, despite the numerous works that gathered data around its coasts for over 150 years. This study brings together and harmonises all macroalgae collections and taxonomical records from multiple projects and teams over the last thirty years.

The result is a list of taxonomic records and georeferenced species occurrence data that substantially increases our knowledge of the coastal Azorean marine biodiversity while providing key information for future ecological studies and to support local, marine management and conservation.

**New information:**

A total of 2283 specimens belonging to 118 taxa of macroalgae are registered here, comprising 61 Rhodophyta, 26 Chlorophyta, 30 Ochrophyta (Phaeophyceae) and one Cyanophyta (Cyanobacteria). From these, 26 taxa are identified only at the genus level and 92 to species level (47 Rhodophyta, 21 Chlorophyta and 24 Ochrophyta). The list comprises 75 native species, 12 records of uncertain origin and five introductions (*Caulerpaprolifera* (Forsskål) J.V.Lamouroux, *Caulerpawebbiana* Montagne, *Hydroclathrustilesii* (Endlicher) Santiañez & M.J.Wynne, *Asparagopsisarmata* Harvey and *Symphyocladiamarchantioides* (Harvey) Falkenberg). *Codiumelisabethiae* O.C.Schmidt represents the only Macaronesian endemism known on the Island. Twenty-three species are newly recorded to the algal flora of the Island.

## Introduction

Despite its isolated position in the middle of the North Atlantic Ocean, the Azores Archipelago holds a diverse marine flora, sharing species with the other Macaronesian archipelagos (Madeira, Savages, Canary and Cabo Verde), the two continental coasts across the North-Atlantic Basin and the Mediterranean Sea (Tittley 2003, Tittley and Neto 2006, Wallenstein et al. 2009). Its flora is considered cosmopolitan and relatively rich when compared to other isolated islands (Neto et al. 2005, Tittley and Neto 2005, Wallenstein et al. 2009). It is the second richest Archipelago in seaweed diversity within Macaronesia, with over 400 recorded species (Freitas 2019) and new records have been added to the known flora after each floristic research (Neto et al. 2022).

With the aim of summarising the present knowledge on macroalgal diversity and occurrences on the Azores, Neto and co-workers recently published a series of data papers (Neto et al. 2020a, Neto et al. 2020b, Neto et al. 2020c, Neto et al. 2021a, Neto et al. 2021b, Neto et al. 2021c) that detail the floristic knowledge throughout the Archipelago (see Table 1). Our work is the latest and last in this series, providing an up-to-date inventory of Faial Island’s marine flora, based upon 30 years of surveys.

Despite recent efforts, the study of marine flora amongst the Azores Islands is not uniform with consequences to the species richness values obtained for the different islands (Table 1). São Miguel, the largest Island in the Archipelago and where an active marine botanical team has been based since the 1980s, leads the list with 323 species and is by far the best studied Island (Neto et al. 2021c).

Our work presents both physical and occurrence data and information gathered from macroalgae surveys undertaken around Faial Island largely by the OKEANOS Centre of the University of the Azores (Link: http://www.okeanos.uac.pt), formerly Department of Oceanography and Fisheries and by the Island Aquatic Research Group of the Azorean Biodiversity Centre of the University of the Azores (Link:  https://ce3c.ciencias.ulisboa.pt/sub-team/island-aquatic-ecology). In these surveys, particular attention was given to the small filamentous and thin sheet-like forms that are often short-lived and fast-growing and usually very difficult to identify without a lab microscope and specialised literature.

The paper aims to provide a valuable marine biological reference for research on systematics, diversity and conservation, biological monitoring, climate change, ecology and other applied studies.

## General description

### Purpose

In this work, we present macroalgae taxonomic records for Faial Island and general information on their occurrence and distribution. By doing so, we are contributing to address several biodiversity shortfalls (see Cardoso et al. 2011, Hortal et al. 2015), namely the need to catalogue the Azorean macroalgae (Linnean shortfall), to improve the current information on their local and regional geographic distribution (Wallacean shortfall) and to understand species abundance patterns and dynamics in space (Prestonian shortfall).

### Additional information

Additional information on these data may be requested from the first author.

## Project description

### Title

Marine algal (seaweed) flora of Faial Island, Azores

### Personnel

Collections and data recording were done by David Milla-Figueras, Ana Isabel Neto, Fernando Tempera, Ignacio Moreu, Afonso C. L. Prestes, Roberto Resendes, Raul M. A. Neto, Ian Tittley, Pedro Afonso, Célia Albuquerque, Norberto Serpa, Gustavo Martins, Maria Vale, Steve Hawkins, Isabel Lima, João Brum, Sara Vanessa Santos, João Gonçalves, Andrea Zita Botelho and Rodrigues Marcos between 1990 and 2018.

Preliminary in situ identifications were carried out by: David Milla-Figueras, Ana Isabel Neto, Fernando Tempera, Sara Vanessa Santos, Afonso C. L. Prestes, Ian Tittley, Gustavo Martins and Andrea Zita Botelho.

David Milla-Figueras, Ana Isabel Neto, Ian Tittley and Fernando Tempera were responsible for the final species identification.

Voucher specimen management was mainly undertaken by David Milla-Figueras, Ana Isabel Neto, Fernando Tempera, Afonso C. L. Prestes, Roberto Resendes, Ian Tittley and Andrea Zita Botelho.

### Study area description

**Figure 1.**
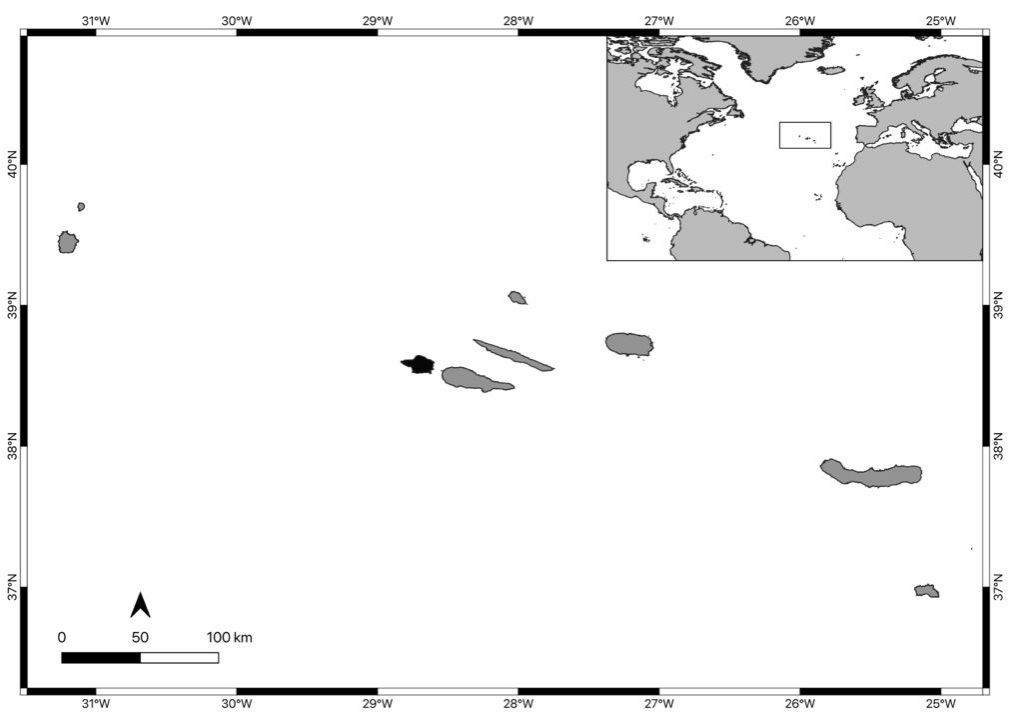
The Azores Archipelago, its location in the North Atlantic and Faial Island highlighted in black.

Faial Island is located at the western end of the Azores’ central island group (38°39′12″N, 28°50′41″W, Fig. 1). The Island is 21 km long along an east-west direction and 14 km wide in a north-south direction, with an area of 173 km². As in the remaining Azorean Islands, the climate is temperate oceanic, with regular and abundant rainfall, high levels of relative humidity and persistent winds, mainly during winter and autumn (Morton et al. 1998). The Island is fringed by a narrow (avg. 2.5 km) shelf that attains its broadest point on the ca. 8 km passage that separates it from the neighbouring Pico Island (Tempera 2008). The tidal range is small (maximum 1.5 m, Instituto Hidrográfico 1981) and coasts are subjected to strong oceanic swells throughout the year. With an extension of approximately 80 km, the island coastline has several bays and small sandy beaches, flanked by rocky cliffs. Intertidal rocky platforms, with variable widths and sometimes dissected by channels and gullies, can be found in a few locations (e.g. Feteira, Capelinhos, Porto da Eira) and are mostly easily accessible from land (authors, pers. observ.).

**Figure 2.**
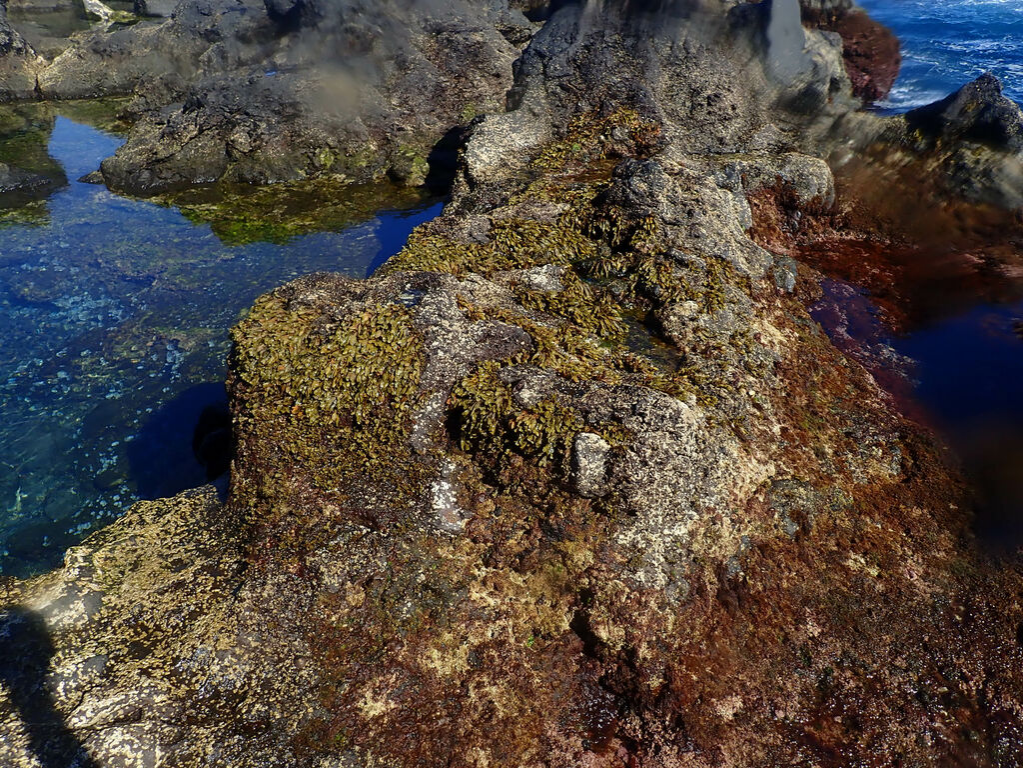
Patches of fringes of the algae *Fucusspiralis* in the upper zone of the mid-littoral.

**Figure 3.**
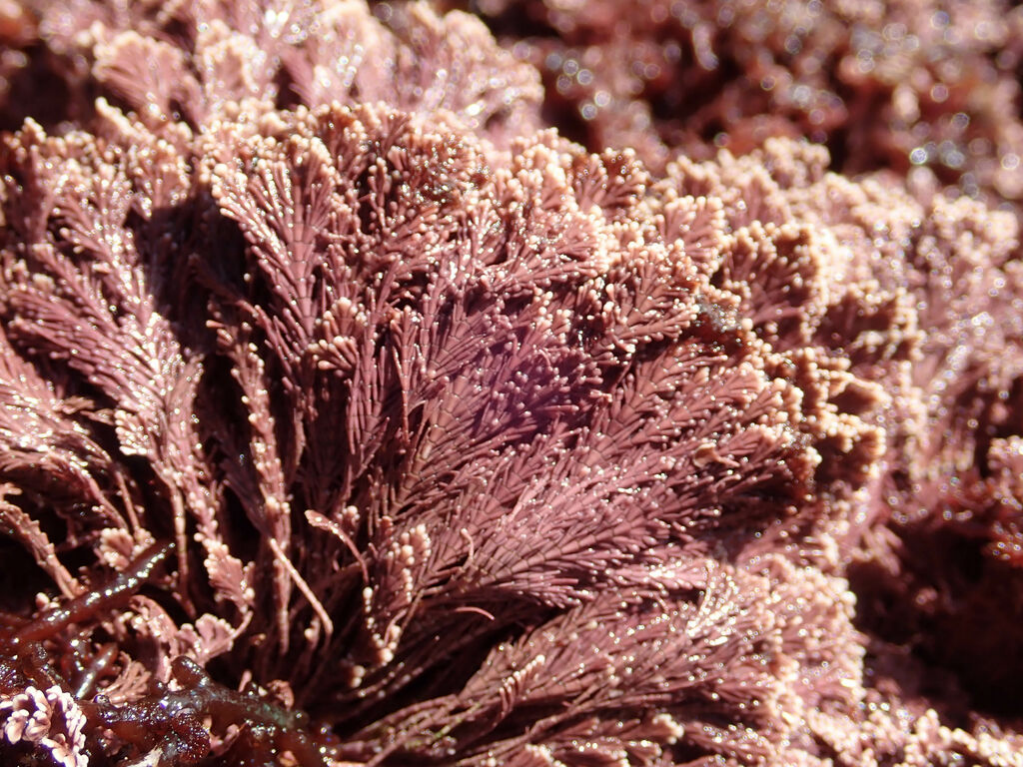
*Corallinaofficinalis* Linnaeus represents one of the common articulated calcareous algae in the mid-littoral zone.

The rocky-shore communities of Faial Island, like all others in the Archipelago, are macroalgae-dominated at both intertidal and shallow subtidal levels (Tittley and Neto 1994, Neto and Tittley 1995). A distinct intertidal zonation pattern is evident with a higher zone dominated by littorinids, followed by the upper zone of the mid-littoral where chthamalid barnacles in which patches of fringes of the algae *Fucusspiralis* Linnaeus and *Gelidiummicrodon* Kützing may occur (Fig. 2). This is followed by the lower zone of the mid-littoral, covered by algal turfs composed either by soft algae (e.g. *Centroceras* sp., *Chondracanthus* sp.) and/or by articulate calcareous ones (e.g. *Corallina* sp. and *Jania* sp., Fig. 3). The lower intertidal zone is mainly dominated by calcareous and non-calcareous encrusting species (notably crustose Corallinaceae and *Codiumadhaerens* C.Agardh) often interspersed with articulated coralline species, such as *Ellisolandiaelongata* (J. Ellis & Solander) K.R.Hind & G.W.Saunders and corticated macrophytes, such as *Pterocladiellacapillacea* (S.G.Gmelin) Santelices & Hommersand (Neto et al. 2005).

**Figure 4.**
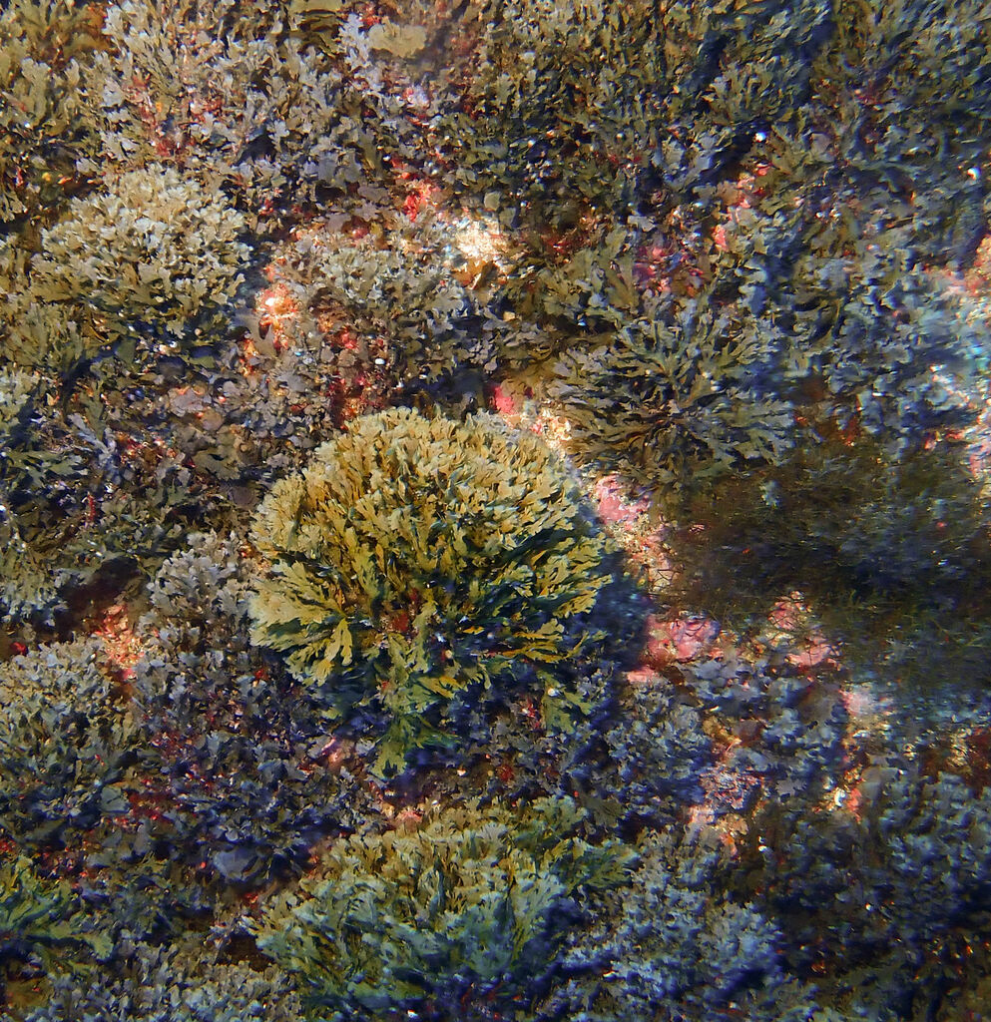
The brown algae *Zonariatournefortii* at the subtidal level.

In the subtidal zone, rocky bottoms are mainly covered by algal turfs where calcareous and/or non-calcareous macroalgae fronds form short, dense multispecific mats (Tempera et al. 2013). The first 10 m are commonly colonised by *Pterocladiellacapillacea*, *Dictyota* spp. and *Halopterisfilicina* (Grateloup) Kützing. Dense stands of *Codiumelisabethiae* are found in sheltered areas between 10 and 25 m depth in the southeast of the Island (Tempera 2008). *Zonariatournefortii* (J.V.Lamouroux) Montagne (Fig. 4) dominates between 15 and 30 m, although facies of *Padina pavonica* (Linnaeus) Thivy can be found in well-lit areas, such as Baixa do Sul. Deeper infra-littoral areas (around 40 m) are often characterised by *Halopterisfilicina* and the rarer *Dictyopterispolypodioides* (De Candolle) J.V.Lamouroux, *Carpomitracostata* (Stackhouse) Batters or *Sporochnuspedunculatus* (Hudson) C.Agardh (Milla-Figueras, pers.observ.).

**Figure 5.**
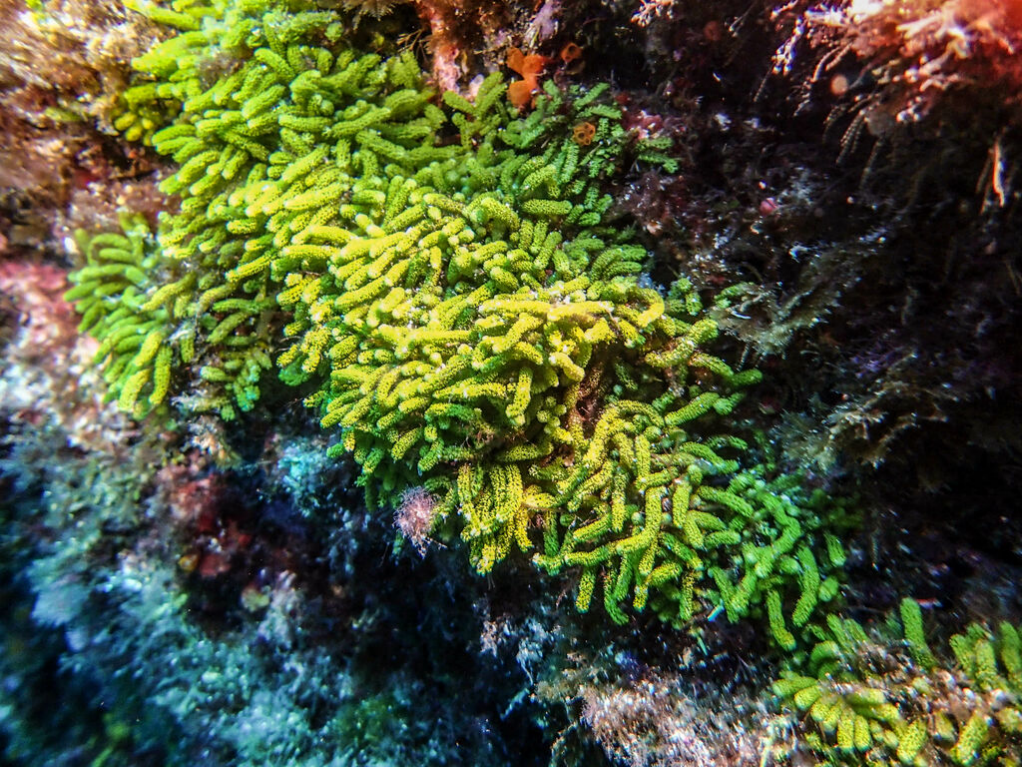
Introduced species *Caulerpawebbiana* on Faial Island's south-eastern coast.

*Caulerpawebbiana* and *C.prolifera* are examples of recent introductions detected over the last two decades (Cardigos et al. 2006, Amat 2008, Cacabelos et al. 2019). *Caulerpawebbiana*, in particular, have been steadily expanding around Faial and neighbouring coasts since it was first recorded (Fig. 5), although, since 2021, it has been replaced by the invasive species *Rugulopteryxokamurae* (E.Y.Dawson) I.K.Hwang, W.J.Lee & H.S.Kim (Milla-Figueras, pers.observ.).

### Design description

**Figure 6.**
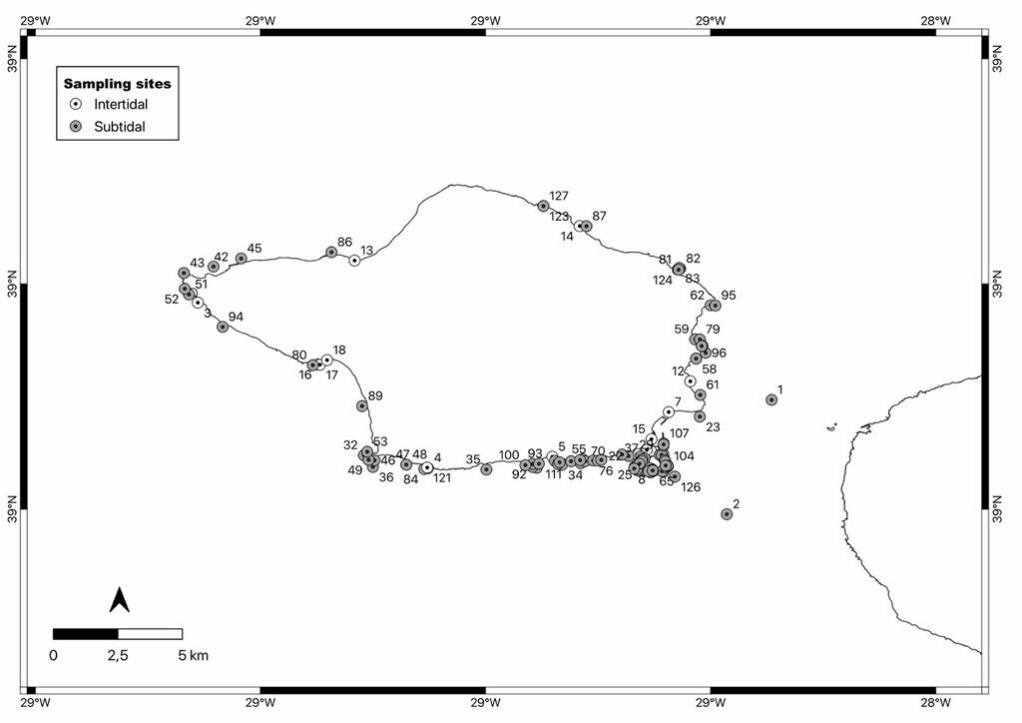
Faial Island showing sampling locations.

The algae were sampled during surveys conducted under various IMAR-DOP/Okeanos projects (1999, 2000, 2002, 2003, 2004, 2005, 2006, 2013, 2015, 2016 and 2018), as well as during a few visits to the Island made by the IBBC-GBA/cE3c group in the years 1990, 1999, 2006 and 2013. Sampling was mainly done by SCUBA divers at subtidal level in various locations spread around the Island and sporadically at the intertidal level (Fig. 6) targeting intertidal platforms at low tide. All specimens collected by the IBBCGBA/cE3c group were given an individual registration number and vouchers were deposited at the AZB Herbarium Ruy Telles Palhinha, based at the Faculty of Sciences and Technology of the University of the Azores. Specimens collected by IMAR-DOP/Okeanos were also stored in vouchers with an individual registration number at the DOP Herbarium, University of the Azores.

### Funding

This study was mainly financed by the following projects/scientific expeditions:

- Portuguese National Funds, through FCT – Fundação para a Ciência e a Tecnologia, within the projects UID/BIA/00329/2019 and UID/BIA/00329/2020-2023;
- Portuguese Regional Funds, through DRCT – Direção Regional da Ciência e Tecnologia, within several projects, since 2013;
- CIRN/DB/UAc (Research Centre for Natural Resources, Universidade dos Açores, Departamento de Biologia);
- CIIMAR (Interdisciplinary Centre of Marine and Environmental Research, Porto, Portugal);
- MARÉ (Life-Nature B4-3200/98/509);
- OGAMP (INTERREG IIIb— MAC/4.2/A2 2001);
- MARMAC (INTERREG IIIb—03/MAC/4.2/A2 2004);
- BALA – “Elaboration of the implementation program of the marine strategy framework directive - biodiversity of the coastal environments of the Azores” (2 /DRAM /2015), funded by the Government of the Azores - Regional Secretariat for the Sea, Science and Technology, Regional Directorate for Sea Affairs, GRA /SRMCT-DRAM;
- MoniZEC-ARP - Monitorização de Áreas Marinhas Protegidas dos Açores com Regulamentação de Restrição à Pesca (30/DRAM/2018), funded by the Government of the Azores - Regional Secretariat for the Sea, Science and Technology, Regional Directorate for Sea Affairs, GRA /SRMCT-DRAM;
- Seaforest Portugal Project (FA_06_2017_067), supported by the Portuguese Government grant Fundo Azul;
- RESTORESEAS – *Marine Forests of animals, plants and algae: nature-based tools to protect and restore biodiversity* (Ref M2.2/RESTORESEAS/002/2022) funded through the joint 2019-2020 Biodiversa & Water JPI joint call for research proposals, under the BiodivRestore ERA-net COFUND programme and with Regional Science and Technology Fund of the Azores (FRCT);
- MoniCO Programme – “Azores Coastal Resources and Environmental Monitoring Program”, SRMCT/DRP. IMAR.

## Sampling methods

### Study extent

Sampling and collections were performed at intertidal and subtidal levels down to approximately 40 m around the Faial Island (Table 2, Fig. 6).

### Sampling description

**Figure 7.**
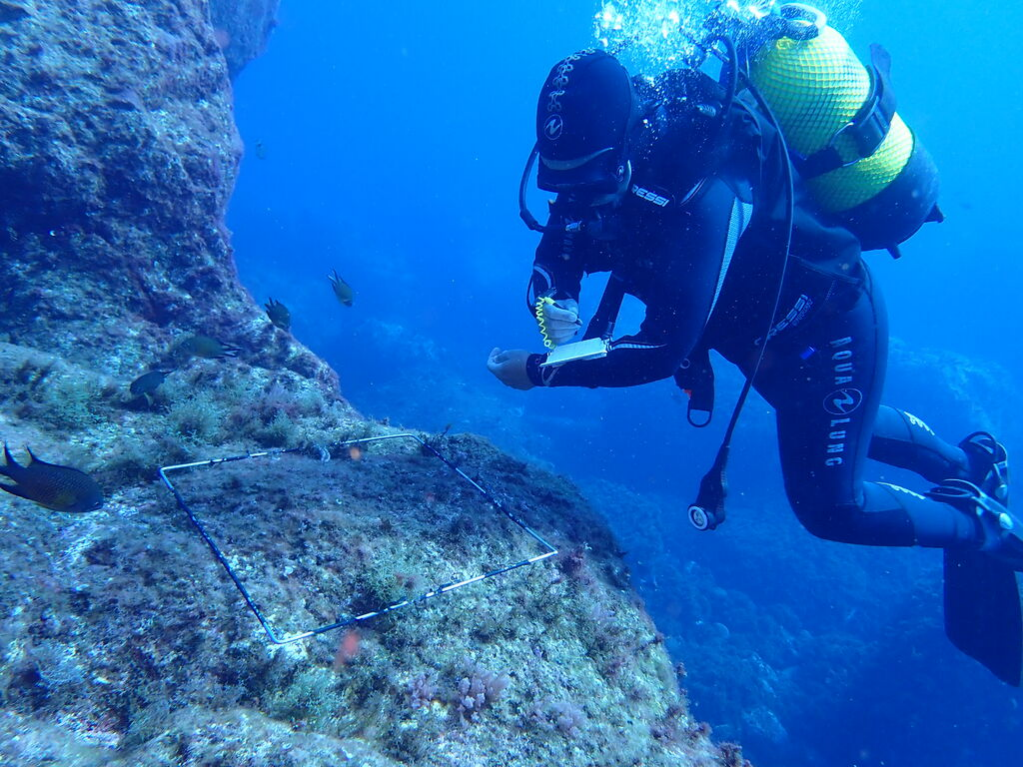
Diver surveying the presence and coverage of macroalgal species on subtidal rocky habitat.

Intertidal collections were made at low tide by walking along the shores. Subtidal collections were made by scuba diving. Sampling encompassed both physical collections and species presence records. For the former, collections were made manually in each sampling location by scraping off one or two specimens of all different species found into previously labelled bags. The species presence records were gathered by registering all species present in the sampling locations visited (Fig. 7). Complementary data, for example, shore level (high, mid, low), orientation, type of substrate (bedrock, boulders, cobbles, mixed) and habitat (tide pool, open rock, gully, crevice, cave) were also recorded.

### Quality control

To identify each sampled taxon we used: 1) morphological observations of the whole specimens with the naked eye and 2) slide preparations examined using dissecting and compound microscopes. This allowed characterisation of diagnosing structures as described in literature and comparison with descriptions from other locations. In more difficult cases, specimens were sent to experts for identification.

### Step description

In the laboratory, the specimens were accommodated, sorted and studied following standard procedures used in macroalgae identification.

Species identification was based on morphological and anatomical characters and reproductive structures. Identifying the small and simple thallus required the observation of the entire thallus with the naked eye and/or using dissecting and compound microscopes. For large and more complex algae, the investigation of the thallus anatomy required histological work to obtain longitudinal and transverse sections needed for the observation of cells, reproductive structures and other diagnosing characteristics.

Various floras and keys from neighbouring territories and biogeographic regions were used for species identification, namely Schmidt 1931, Taylor 1967, Levring 1974, Dixon and Irvine 1977, Taylor 1978, Lawson and John 1982, Anonymous 1983, Gayral and Cosson 1986, Fletcher 1987, Afonso-Carrillo and Sansón 1989, Burrows 1991, Boudouresque et al. 1992, Cabioc'h et al. 1992, Maggs and Hommersand 1993, Irvine and Chamberlain 1994, Brodie et al. 2007, Lloréns et al. 2012, Rodríguez-Prieto et al. 2013.

For more critical and taxonomically difficult taxa, specimens were taken to the Natural History Museum (London) for comparison with collections there or sent to appropriate specialists.

A reference collection was made for all specimens collected by giving them a herbarium code number and depositing them at the AZB Herbarium Ruy Telles Palhinha, University of Azores. Depending on the species and further planned research, different types of collections were made, namely: (i) liquid collections using 5% buffered formaldehyde seawater and then replacing it with Kew (Bridsen and Forman 1999); (ii) dried collections, either by pressing the algae (most species) following the method described by Gayral and Cosson (1986) or by letting them air dry (calcareous species); and (iii) silica collections.

Nomenclatural and taxonomic status used here follow Algaebase (Guiry and Guiry 2021). The database was organised on FileMaker Pro.

## Geographic coverage

### Description

Faial Island, Azores, Macaronesia, Portugal (approximately 38°39′12″N 28°50′41″W).

### Coordinates

38.489 and 38.653 Latitude; -28.845 and -28.5589 Longitude.

## Taxonomic coverage

### Description

All macroalgae were identified to genus or species level. In total, 118 taxa were identified belonging to 24 orders and 50 families, distributed by the phyla Rhodophyta (12 orders and 31 families), Chlorophyta (4 orders and 8 families), Ochrophyta (7 orders and 10 families) and Cyanobacteria (1 order and 1 family).

### Taxa included

**Table:** 

Rank	Scientific Name	Common Name
phylum	Rhodophyta	Red algae
phylum	Chlorophyta	Green algae
kingdom	Ochrophyta	Brown algae
kingdom	Cyanobacteria	Cyanophyta

## Temporal coverage

### Notes

The sampling was performed on multiple fields studied between 1990 and 2018.

## Collection data

### Collection name

AZB|Marine macroalgae collection of Faial Island-Occasional sampling; DOP|Marine macroalgae occurrence of Faial Island (Azores)-Project BALA; DOP|Marine macroalgae occurrence of Faial Island (Azores)-Project MARÉ; DOP|Marine macroalgae occurrence of Faial Island (Azores)-Project MARMAC; DOP|Marine macroalgae occurrence of Faial Island (Azores)-Project MONIZEC-ARP; DOP|Marine macroalgae occurrence of Faial Island (Azores)-Project OGAMP; DOP|Marine macroalgae collection of Faial Island-Occasional sampling.

### Collection identifier

9fdfcab6-69d9-45bc-8210-b9b91eef474f; 1b1bd2dc-3c74-4467-b620-b663789826b5; 2aad17d8-428d-42ca-b22d-ab51c5dbcd2b; e0e7c8f9-56c4-4302-8db3-45e52af9f822; 0ac2d400-8e09-4d70-9f4c-a7d1ee50b478; ce6485b1-82ed-44ea-80e4-276fa4aee65b; a791eabf-5003-492d-be2e-69d921f9840f

### Parent collection identifier

AZB Herbarium Ruy Telles Palhinha, Faculty of Sciences and Technology of the University of the Azores; n/a; n/a; n/a; n/a; n/a; DOP Herbarium, Faculty of Sciences and Technology of the University of the Azores.

### Specimen preservation method

Specimens were preserved as follows: air-dry, dried and pressed; liquid (Formalin; Alcohol), silica.

### Curatorial unit

AZB Herbarium Ruy Telles Palhinha, Faculty of Sciences and Technology of the University of the Azores and DOP Herbarium, Faculty of Sciences and Technology of the University of the Azores.

## Usage licence

### Usage licence

Creative Commons Public Domain Waiver (CC-Zero)

## Data resources

### Data package title

Marine algal (seaweed) flora of Faial Island, Azores

### Resource link

https://doi.org/10.15468/dsajcw

### Alternative identifiers

https://www.gbif.org/dataset/a5650ad0-caa5-4d2d-ad71-cd24356df369; http://ipt.gbif.pt/ipt/resource?r=faial_seaweed

### Number of data sets

2

### Data set 1.

#### Data set name

Marine algal (seaweed) flora of Faial Island, Azores

#### Data format

Darwin Core Archive

#### Character set

UTF-8

#### Download URL

http://ipt.gbif.pt/ipt/resource?r=faial_seaweed

#### Data format version

1.11

#### Description

The dataset table includes all the records for which a taxonomic identification of the species was possible. The dataset submitted to GBIF is structured as a sample event dataset, with two tables: event (as core) and occurrences (abundance data). The data in this sampling event resource have been published as a Darwin Core Archive (DwCA), a standardised format for sharing biodiversity data as a set of one or more data tables. The core data file contains 162 records (eventID). The data and resource metadata are available for download from Milla-Figueras et al. (2024).

**Data set 1.** 

Column label	Column description
eventID	Identifier of the event, unique for the dataset.
country	Country of the sampling site.
country code	Code of the country where the event occurred.
stateProvince	Name of the region.
island	Name of the island.
municipality	Name of the municipality.
locality	Name of the locality.
locationID	Identifier of the location.
decimalLatitude	The geographic latitude of the sampling site.
decimalLongitude	The geographic longitude of the sampling site.
geodeticDatum	The spatial reference system upon which the geographic coordinates are based.
eventDate	Time interval when the event occurred.
Year	The year of the event.
samplingProtocol	Sampling method used during an event.
locationRemarks	Zonation level.
minimumDepthInMetres	The minimum depth in metres where the specimen was found.
maximumDepthInMetres	The maximum depth in metres where the specimen was found.
eventRemarks	Notes about the event.

### Data set 2.

#### Data set name

Marine algal (seaweed) flora of Faial Island, Azores

#### Data format

Darwin Core Archive

#### Character set

UTF-8

#### Download URL

http://ipt.gbif.pt/ipt/resource?r=faial_seaweed

#### Data format version

1.11

#### Description

The dataset table includes all the records for which a taxonomic identification of the species was possible. The dataset submitted to GBIF is structured as a sample event dataset, with two tables: event (as core) and occurrences (abundance data). The data in this sampling event resource have been published as a Darwin Core Archive (DwCA), a standardised format for sharing biodiversity data as a set of one or more data tables. The core data file contains 2283 records (occurrenceID). The data and resource metadata are available for download from Milla-Figueras et al. (2024).

**Data set 2.** 

Column label	Column description
occurrenceID	Identifier of the record, coded as a global unique identifier.
institutionID	The identifier for the institution having custody of the object or information referred to in the record.
institutionCode	The acronym of the institution having custody of the object or information referred to in the record.
collectionID	An identifier of the collection to which the record belongs.
collectionCode	The name of the collection from which the record was derived.
datasetName	The name identifying the dataset from which the record was derived.
eventID	Identifier of the event, unique for the dataset.
kingdom	Kingdom name.
phylum	Phylum name.
class	Class name.
order	Order name.
family	Family name.
genus	Genus name.
scientificName	The name without authorship applied on the first identification of the specimen.
establishmentMeans	Statement about whether a dwc:Organism has been introduced to a given place and time through the direct or indirect activity of modern humans.
scientificNameAuthorship	The authorship information for the scientificName formatted according to the conventions of the applicable nomenclatureCode.
acceptedNameUsage	The specimen accepted name, with authorship.
previousIdentifications	Previous name of the specimen, with authorship.
basisOfRecord	The specific nature of the data record.
habitat	Description of the habitat where the specimen was found.
recordedBy	Person(s) responsible for sampling.
catalogNumber	Identifying code for a unique sample lot in a biological collection.
identifiedBy	Person(s) responsible for taxa identification.
type	The nature of the resource.
preparations	The preservation method used for the specimen.
establishmentMeans	The establishment status of the organism in the study region.
ocurrenceRemarks	New record status assignment.
licence	Reference to the licence under which the record is published.

## Additional information

This paper is based on 2283 specimens of macroalgae recorded from Faial Island in 118 taxa, comprising 92 confirmed species (Table 3) and 26 taxa identified only to genus level. The confirmed species (Table 4) include 47 Rhodophyta, 21 Chlorophyta and 24 Ochrophyta (Phaeophyceae). Of these, twenty-three species are newly recorded to the Island (12 Rhodophyta, 3 Chlorophyta and 8 Ochrophyta). Most species (75) are native, including *Codiumelisabethiae*, the only Macaronesian endemic known in the Island. Twelve have an uncertain status (7 Rhodophyta, 3 Chlorophyta and 2 Ochrophyta) and five species represent introductions to the algal flora (*Caulerpaprolifera*, *Caulerpawebbiana*, *Hydroclathrustilesii*, *Asparagopsisarmata* and *Symphyocladiamarchantioides*).

Six species were commonly found around the Island with a high relative abundance, the Ochrophyta species *Dictyotadichotoma* (Hudson) J.V.Lamouroux, *Halopterisfilicina*, *Lobophoravariegata* (J.V.Lamouroux) Womersley ex E.C.Oliveira, *Padina pavonica* and *Zonariatournefortii*; as well as the Rhodophyta *Asparagopsistaxiformis* (Delile) Trevisan. Many other species were only sporadically found, with few records or just a single record, as the case of the non-indigenous species *Caulerpaprolifera* recorded just once in a pool of the north of the Island.

## Figures and Tables

**Table 1.** Number of macroalgal species on the Azores Islands (Neto et al. 2020a, Neto et al. 2020b, Neto et al. 2020c, Neto et al. 2021a, Neto et al. 2021b, Neto et al. 2021c and authors' unpublished data).

Phyllum	Santa Maria	São Miguel	Terceira	Graciosa	São Jorge	Pico	Faial	Flores	Corvo
Rhodophyta	102	212	73	126	35	142	59	80	22
Chlorophyta	29	48	24	31	17	41	16	22	8
Ochrophyta	44	63	16	38	10	42	8	26	13
Total	175	323	113	195	62	225	83	128	43

**Table 2.** Information and location of sampling sites on Faial Island.

Location N	Location ID	Municipality	Locality	Latitude	Longitude	Littoral zone
1	FAI_H_BN	Horta	Baixa do Norte	38.548503	-28.572914	Subtidal
2	FAI_H_BS	Horta	Baixa do Sul	38.497737	-28.592802	Subtidal
3	FAI_H_Cpc	Horta	Capelinhos|Porto Comprido	38.591696	-28.82786	Intertidal
4	FAI_H_CBpi	Horta	Castelo Branco|Piscinas	38.518186	-28.725545	Intertidal
5	FAI_H_Fp	Horta	Feteira|Porto	38.52325	-28.67036	Intertidal
6	FAI_H_Hpd	Horta	Horta|Ponta da Doca	38.524268	-28.620946	Subtidal
7	FAI_H_Hpa	Horta	Horta|Praia da Alagoa	38.543115	-28.618633	Intertidal
8	FAI_H_MGbi	Horta	Monte da Guia|Baía interna	38.517818	-28.626165	Intertidal
9	FAI_H_PPbem	Horta	Porto Pim|Baía|Entre-marés	38.5258827	-28.626551	Intertidal
10	FAI_H_Pem	Horta	Pasteleiro|Entre-marés	38.524456	-28.632701	Intertidal
11	FAI_H_PPr	Horta	Porto Pim|Rampa de varagem	38.5263	-28.628352	Intertidal
12	FAI_H_PA	Horta	Praia do Almoxarife	38.556763	-28.609026	Intertidal
13	FAI_H_PNf	Horta	Praia do Norte|Fajã	38.610399	-28.7582	Intertidal
14	FAI_H_Sp	Horta	Salão|Porto	38.625731	-28.658197	Intertidal
15	FAI_H_SCc	Horta	Santa Cruz|Cais	38.531075	-28.626295	Intertidal
16	FAI_H_Vpic	Horta	Varadouro|Piscina|canal	38.564297	-28.774541	Intertidal
17	FAI_H_Vp	Horta	Varadouro|Porto	38.564141	-28.773566	Intertidal
18	FAI_H_Vpr	Horta	Varadouro|Porto|rampa de varagem	38.566167	-28.770433	Intertidal
19	FAI_H_BC	Horta	Baía da Cabra	38.516795	-28.630027	Subtidal
20	FAI_H_PFF	Horta	Ponta Furada (fora)	38.520394	-28.657772	Subtidal
21	FAI_H_CF	Horta	Caldeirinha de Fora	38.516292	-28.626644	Subtidal
22	FAI_H_P	Horta	Pasteleiro	38.52359	-28.636438	Subtidal
23	FAI_H_E	Horta	Espalamaca	38.541091	-28.604817	Subtidal
24	FAI_H_IPP	Horta	Ilhéus de Porto Pim	38.518833	-28.63373	Subtidal
25	FAI_H_IPS	Horta	Ilhéu Pequeno S	38.519819	-28.632242	Subtidal
26	FAI_H_IPW	Horta	Ilhéu Pequeno W	38.519839	-28.632567	Subtidal
27	FAI_H_IPN	Horta	Ilhéu Pequeno N	38.520167	-28.632217	Subtidal
28	FAI_H_IN	Horta	Ilhéu Negro	38.520961	-28.631222	Subtidal
29	FAI_H_BE	Horta	Baía de Entre-Montes	38.524084	-28.622656	Subtidal
30	FAI_H_TA	Horta	Tomates do Almeida	38.517137	-28.632363	Subtidal
31	FAI_H_FSS	Horta	Forte de S. Sebastião	38.523447	-28.630946	Subtidal
32	FAI_H_MCB	Horta	Morro de Castelo Branco	38.523923	-28.754064	Subtidal
33	FAI_H_CD	Horta	Caldeirinha de Dentro	38.518333	-28.6275	Subtidal
34	FAI_H_FE	Horta	Feteira Este	38.519683	-28.66605	Subtidal
35	FAI_H_CBA	Horta	Castelo Branco: Aeroporto	38.517571	-28.699626	Subtidal
36	FAI_H_BCB	Horta	Baixa de Castelo Branco	38.518746	-28.750053	Subtidal
37	FAI_H_PP	Horta	Porto Pim	38.522551	-28.629359	Subtidal
38	FAI_H_PRN	Horta	Ponta dos Radares (N)	38.51907	-28.619117	Subtidal
39	FAI_H_MGL	Horta	Monte da Guia: Costa Leste, à saída da furna Norte	38.516558	-28.620925	Subtidal
40	FAI_H_PG	Horta	Ponta das Garças	38.517778	-28.632684	Subtidal
41	FAI_H_BF	Horta	Baixa da Feteira	38.518397	-28.677222	Subtidal
42	FAI_H_CNE2	Horta	Capelinhos: Limite Nordeste do SIC	38.601133	-28.8086	Subtidal
43	FAI_H_CPW	Horta	Capelinhos (Ponta W, Porta dos Piratas)	38.60485	-28.834033	Subtidal
44	FAI_H_CNW	Horta	Capelinhos: Ponta NW	38.602862	-28.836063	Subtidal
45	FAI_H_CNE1	Horta	Capelinhos: Limite NE do SIC entre o Ilhéu e a zona dos paralelipípedos	38.611333	-28.8086	Subtidal
46	FAI_H_MCBLE	Horta	Morro de Castelo Branco (lado Este)	38.5217	-28.749333	Subtidal
47	FAI_H_MCBEL	Horta	Morro de Castelo Branco (extremo Leste, limite do SIC)	38.519733	-28.735217	Subtidal
48	FAI_H_MCBFL	Horta	Morro de Castelo Branco (Fora do Limite Este do SIC)	38.519169	-28.730413	Subtidal
49	FAI_H_MCBFS	Horta	Morro de Castelo Branco - Face Sul	38.521902	-28.75166	Subtidal
50	FAI_H_MCBZS	Horta	Morro de Castelo Branco - Zona Sul	38.521883	-28.75195	Subtidal
51	FAI_H_IZS	Horta	Ilhéu da Zona Sul do SIC dos Capelinhos	38.595317	-28.831767	Subtidal
52	FAI_H_CS	Horta	Capelinhos (lado Sul)	38.59785	-28.83365	Subtidal
53	FAI_H_MCBNE	Horta	Morro de Castelo Branco (NE)	38.525467	-28.752583	Subtidal
54	FAI_H_BR	Horta	Baía dos Radares	38.518475	-28.620303	Subtidal
55	FAI_H_PF	Horta	Ponta Furada	38.522153	-28.656906	Subtidal
56	FAI_H_PPM	Horta	Porto de Pedro Miguel	38.566852	-28.606463	Subtidal
57	FAI_H_PPMN	Horta	Porto de Pedro Miguel N	38.567175	-28.605564	Subtidal
58	FAI_H_PPMSW	Horta	Porto de Pedro Miguel (rochas a sw do portinho)	38.566795	-28.606956	Subtidal
59	FAI_H_PM	Horta	Pedro Miguel (na restinga a N da cascata)	38.5754	-28.606883	Subtidal
60	FAI_H_MGE	Horta	Monte da Guia: costa Leste gruta N	38.516847	-28.621633	Subtidal
61	FAI_H_EN	Horta	Espalamaca (N)	38.550727	-28.6046	Subtidal
62	FAI_H_RP	Horta	Ribeirinha (porto)	38.5905	-28.60005	Subtidal
63	FAI_H_CF1	Horta	Caldeirinha de fora (Replic.1)	38.517367	-28.626085	Subtidal
64	FAI_H_CF2	Horta	Caldeirinha de fora (Replic.2)	38.517267	-28.625733	Subtidal
65	FAI_H_CF3	Horta	Caldeirinha de fora (Replic.3)	38.51695	-28.625483	Subtidal
66	FAI_H_BEM2	Horta	Baía de Entre-Montes	38.522167	-28.620633	Subtidal
67	FAI_H_BEMD	Horta	Baía de Entre-Montes (dentro)	38.523211	-28.623616	Subtidal
68	FAI_H_C4	Horta	Caldeirinhas 4	38.5171	-28.62685	Subtidal
69	FAI_H_BEM3	Horta	Baía de Entre-Montes	38.524025	-28.621781	Subtidal
70	FAI_H_PF1	Horta	Ponta Furada 1	38.52125	-28.655817	Subtidal
71	FAI_H_C5	Horta	Caldeirinhas 5	38.5171	-28.62685	Subtidal
72	FAI_H_C6	Horta	Caldeirinhas 6	38.5171	-28.62685	Subtidal
73	FAI_H_PR	Horta	Ponta dos Radares	38.518517	-28.619683	Subtidal
74	FAI_H_PPIN2	Horta	Porto Pim - Ilhéu Negro 2	38.5215	-28.630917	Subtidal
75	FAI_H_P1	Horta	Pasteleiro 1	38.52425	-28.63935	Subtidal
76	FAI_H_P2	Horta	Pasteleiro 2	38.521567	-28.652217	Subtidal
77	FAI_H_P3	Horta	Pasteleiro 3	38.521517	-28.650133	Subtidal
78	FAI_H_P4	Horta	Pasteleiro 4	38.521883	-28.648617	Subtidal
79	FAI_H_PMN	Horta	Pedro Miguel N	38.575367	-28.604783	Subtidal
80	FAI_H_VW	Horta	Varadouro (Baía a w das piscinas)	38.563933	-28.776733	Subtidal
81	FAI_H_CNF1	Horta	Costa N Faial (inhames)	38.6064	-28.6146	Subtidal
82	FAI_H_CNF2	Horta	Costa N Faial (inhames)	38.606817	-28.613583	Subtidal
83	FAI_H_CNF3	Horta	Costa N Faial (inhames)	38.6071	-28.614	Subtidal
84	FAI_H_CBW	Horta	Castelo Branco: a W das Piscinas	38.517817	-28.727217	Subtidal
85	FAI_H_PPE	Horta	Porto Pim - E	38.522117	-28.630367	Subtidal
86	FAI_H_PNL	Horta	Praia do Norte (lixeira)	38.614117	-28.76845	Subtidal
87	FAI_H_SE	Horta	Salão E	38.625656	-28.655194	Subtidal
88	FAI_H_F4	Horta	Feteira	38.521367	-28.669167	Subtidal
89	FAI_H_V2	Horta	Varadouro 2	38.545783	-28.754833	Subtidal
90	FAI_H_IF	Horta	Ilhéus da Feteira	38.518733	-28.679167	Subtidal
91	FAI_H_BEM1	Horta	Baía de Entre-Montes	38.521817	-28.620417	Subtidal
92	FAI_H_F2	Horta	Feteira	38.520033	-28.679117	Subtidal
93	FAI_H_F3	Horta	Feteira	38.520167	-28.6763	Subtidal
94	FAI_H_FVF	Horta	Farol do Vale Formoso	38.580933	-28.816833	Subtidal
95	FAI_H_R	Horta	Ribeirinha	38.590414	-28.597903	Subtidal
96	FAI_H_PM1	Horta	Pedro Miguel	38.569417	-28.602217	Subtidal
97	FAI_H_PM2	Horta	Pedro Miguel	38.572483	-28.60365	Subtidal
98	FAI_H_PF2	Horta	Ponta Furada	38.52125	-28.662017	Subtidal
99	FAI_H_PF3	Horta	Ponta Furada	38.521767	-28.658017	Subtidal
100	FAI_H_F1	Horta	Feteira	38.5195	-28.6823	Subtidal
101	FAI_H_IN3	Horta	Ilhéu Negro	38.5211833	-28.630933	Subtidal
102	FAI_H_IN2	Horta	Ilhéu Negro	38.5202333	-28.631483	Subtidal
103	FAI_H_R2	Horta	Radares	38.5194833	-28.6195	Subtidal
104	FAI_H_R1	Horta	Radares	38.5190333	-28.618983	Subtidal
105	FAI_H_IN4	Horta	Ilhéu Negro	38.52135	-28.630583	Subtidal
106	FAI_H_IN1	Horta	Ilhéu Negro	38.5201833	-28.631617	Subtidal
107	FAI_H_D3	Horta	Doca	38.5291333	-28.620883	Subtidal
108	FAI_H_D1	Horta	Doca	38.5281333	-28.620983	Subtidal
109	FAI_H_D2	Horta	Doca	38.5287167	-28.620933	Subtidal
110	FAI_H_CF4	Horta	Caldeirinha de Fora	38.5168333	-28.627633	Subtidal
111	FAI_H_L1	Horta	Lajinha	38.52005	-28.667867	Subtidal
112	FAI_H_L2	Horta	Lajinha	38.5208	-28.667117	Subtidal
113	FAI_H_BEM	Horta	Baia Entremontes	38.52115	-28.620117	Subtidal
114	FAI_H_R3	Horta	Radares	38.5190838	-28.619316	Subtidal
115	FAI_H_R6	Horta	Radares	38.5193386	-28.619061	Subtidal
116	FAI_H_R4	Horta	Radares	38.5192113	-28.619189	Subtidal
117	FAI_H_R5	Horta	Radares	38.5192486	-28.619151	Subtidal
118	FAI_H_CF6	Horta	Caldeirinha de Fora	38.5171817	-28.625752	Subtidal
119	FAI_H_CF5	Horta	Caldeirinha de Fora	38.5171667	-28.625767	Subtidal
120	FAI_H_BR2	Horta	Baía dos Radares2	38.519333	-28.62	Subtidal
121	FAI_H_CB	Horta	Castelo Branco	38.518468	-28.725933	Intertidal
122	FAI_H_BQ	Horta	Baía Quadrada	38.518	-28.634	Subtidal
123	FAI_H_PE	Horta	Porto da Eira	38.63461	-28.674311	Intertidal
124	FAI_H_CRS	Horta	Costa Ribeirinha-Salão	38.606299	-28.614249	Subtidal
125	FAI_H_PM3	Horta	Pedro Miguel	38.572441	-28.603977	Subtidal
126	FAI_H_MGF	Horta	Fora do Monte da Guia	38.514412	-28.615878	Subtidal
127	FAI_H_PE2	Horta	Porto da Eira	38.6346	-28.674314	Subtidal

**Table 3.** Macroalgal species recorded from Faial Island, with information on relative abundance, origin and status.

Phyllum	Species (Accepted Name)	Number of records	Establishment Means	Occurrence Remarks
Chlorophyta	*Anadyomenestellata* (Wulfen) J.Agardh	25	Uncertain	
Chlorophyta	*Caulerpaprolifera* (Forsskål) J.V.Lamouroux	1	Introduced	New record
Chlorophyta	*Caulerpawebbiana* Montagne	61	Introduced	
Chlorophyta	*Chaetomorphaaerea* (Dillwyn) Kützing	1	Native	
Chlorophyta	*Chaetomorphapachynema* (Montagne) Kützing	1	Native	
Chlorophyta	*Cladophoraalbida* (Nees) Kützing	1	Native	
Chlorophyta	*Cladophoraprolifera* (Roth) Kützing	1	Native	
Chlorophyta	*Cladophoropsismembranacea* (Bang ex C.Agardh) Børgesen	1	Uncertain	
Chlorophyta	*Codiumadhaerens* C.Agardh	26	Native	
Chlorophyta	*Codiumdecorticatum* (Woodward) M.A.Howe	1	Native	
Chlorophyta	*Codiumelisabethiae* O.C.Schmidt	67	Macaronesian endemism	
Chlorophyta	*Codiumtomentosum* Stackhouse	11	Native	
Chlorophyta	*Codiumvermilara* (Olivi) Delle Chiaje	1	Native	New record
Chlorophyta	*Microdictyonumbilicatum* (Velley) Zanardini	27	Native	
Chlorophyta	*Ulvaclathrata* (Roth) C.Agardh	1	Native	
Chlorophyta	*Ulvaintestinalis* Linnaeus	1	Native	
Chlorophyta	*Ulvalactuca* Linnaeus	1	Uncertain	
Chlorophyta	*Ulvalinza* Linnaeus	1	Native	
Chlorophyta	*Ulvaprolifera* O.F.Müller	1	Native	New record
Chlorophyta	*Ulvarigida* C.Agardh	3	Native	
Chlorophyta	*Valoniautricularis* (Roth) C.Agardh	20	Native	
Ochrophyta	*Ascophyllumnodosum* (Linnaeus) Le Jolis	3	Native	
Ochrophyta	*Carpomitracostata* (Stackhouse) Batters	1	Native	
Ochrophyta	*Cladostephusspongiosus* (Hudson) C.Agardh	40	Native	
Ochrophyta	*Colpomeniasinuosa* (Mert. ex Roth) Derbès & Solier	15	Native	
Ochrophyta	*Cutleriamultifida* (Turner) Greville phase Aglaozonia parvula (Greville) Zanardini	40	Uncertain	
Ochrophyta	*Cystoseirahumilis* Schousboe ex Kützing	1	Native	New record
Ochrophyta	*Dictyopterispolypodioides* (De Candolle) J.V.Lamouroux	1	Native	New record
Ochrophyta	*Dictyotaciliolata* Sonder ex Kützing	1	Native	New record
Ochrophyta	*Dictyotadichotoma* (Hudson) J.V.Lamouroux	129	Native	
Ochrophyta	*Fucusspiralis* Linnaeus	2	Uncertain	New record
Ochrophyta	*Gongolariaabies-marina* (S.G.Gmelin) Kuntze	40	Native	
Ochrophyta	*Halopterisfilicina* (Grateloup) Kützing	143	Native	
Ochrophyta	*Halopterisscoparia* (Linnaeus) Sauvageau	86	Native	
Ochrophyta	*Hydroclathrustilesii* (Endlicher) Santiañez & M.J.Wynne	1	Introduced	New record
Ochrophyta	*Lobophoravariegata* (J.V.Lamouroux) Womersley ex E.C.Oliveira	142	Native	
Ochrophyta	*Nemodermatingitanum* Schousboe ex Bornet	15	Native	
Ochrophyta	*Padina pavonica* (Linnaeus) Thivy	126	Native	
Ochrophyta	*Petrospongiumberkeleyi* (Greville) Nägeli ex Kützing	1	Native	
Ochrophyta	*Sargassumcymosum* C.Agardh	1	Native	
Ochrophyta	*Sargassumnatans* (Linnaeus) Gaillon	1	Native	New record
Ochrophyta	*Sporochnuspedunculatus* (Hudson) C.Agardh	1	Native	New record
Ochrophyta	*Stypopodiumzonale* (J.V.Lamouroux) Papenfuss	1	Native	New record
Ochrophyta	*Taoniaatomaria* (Woodward) J.Agardh	37	Native	
Ochrophyta	*Zonariatournefortii* (J.V.Lamouroux) Montagne	101	Native	
Rhodophyta	*Acrosoriumciliolatum* (Harvey) Kylin	9	Native	New record
Rhodophyta	*Acrosymphytonpurpuriferum* (J.Agardh) G.Sjöstedt	2	Native	
Rhodophyta	*Amphiroabeauvoisii* J.V.Lamouroux	2	Native	
Rhodophyta	*Asparagopsisarmata* Harvey	89	Introduced	
Rhodophyta	*Asparagopsistaxiformis* (Delile) Trevisan	113	Native	
Rhodophyta	*Asteromeniapeltata* (W.R.Taylor) Huisman & A.J.K.Millar	1	Native	New record
Rhodophyta	*Callithamniontetragonum* (Stackhouse) S.F.Gray	1	Native	
Rhodophyta	*Caulacanthusustulatus* (Turner) Kützing	1	Uncertain	
Rhodophyta	*Centrocerasclavulatum* (C.Agardh) Montagne	1	Native	
Rhodophyta	*Ceramiumdeslongchampsii* Chauvin ex Duby	1	Native	New record
Rhodophyta	*Champiaparvula* (C.Agardh) Harvey	6	Native	
Rhodophyta	*Chondracanthusacicularis* (Roth) Fredericq	8	Native	
Rhodophyta	*Chondriadasyphylla* (Woodward) C.Agardh	2	Uncertain	
Rhodophyta	*Corallinaofficinalis* Linnaeus	6	Native	
Rhodophyta	*Cottoniellafilamentosa* (M.Howe) Børgesen	6	Native	New record
Rhodophyta	*Dermocorynusdichotomus* (J.Agardh) Gargiulo, Morabito & Manghisi	2	Native	
Rhodophyta	*Ellisolandiaelongata* (J.Ellis & Solander) K.R.Hind & G.W.Saunders	30	Native	
Rhodophyta	*Erythrodermistraillii* (Holmes ex Batters) Guiry & Garbary	1	Uncertain	
Rhodophyta	*Gelidiummicrodon* Kützing	3	Native	
Rhodophyta	*Gelidiumspinosum* (S.G.Gmelin) P.C.Silva	2	Native	
Rhodophyta	*Grateloupiafilicina* (J.V.Lamour.) C.Agardh	2	Native	New record
Rhodophyta	*Gymnogongruscrenulatus* (Turner) J.Agardh	3	Native	
Rhodophyta	*Halarachnionligulatum* (Woodward) Kützing	4	Native	New record
Rhodophyta	*Herposiphoniasecunda* (C.Agardh) Ambronn	2	Native	
Rhodophyta	*Hypneamusciformis* (Wulfen) J.V.Lamouroux	12	Uncertain	
Rhodophyta	*Hypneaspinella* (C.Agardh) Kützing	2	Native	New record
Rhodophyta	*Janiacrassa* J.V.Lamouroux	2	Native	
Rhodophyta	*Janiarubens* (Linnaeus) J.V.Lamouroux	2	Native	
Rhodophyta	*Janiavirgata* (Zanardini) Montagne	4	Uncertain	
Rhodophyta	*Laurenciaobtusa* (Hudson) J.V.Lamouroux	2	Native	New record
Rhodophyta	*Leptosiphoniabrodiei* (Dillwyn) A.M.Savoie & G.W.Saunders	2	Uncertain	
Rhodophyta	*Liagoradistenta* (Mertens ex Roth) J.V.Lamouroux	1	Native	New record
Rhodophyta	*Lomentariaarticulata* (Hudson) Lyngbye	1	Native	
Rhodophyta	*Meredithiamicrophylla* (J.Agardh) J.Agardh	3	Native	New record
Rhodophyta	*Neopyropialeucosticta* (Thuret) L.-E.Yang & J.Brodie	2	Native	New record
Rhodophyta	*Nitophyllumpunctatum* (Stackhouse) Greville	10	Native	
Rhodophyta	*Osmundeahybrida* (A.P.de Candolle) K.W.Nam	4	Native	
Rhodophyta	*Osmundeapinnatifida* (Hudson) Stackhouse	3	Native	
Rhodophyta	*Plocamiumcartilagineum* (Linnaeus) P.S.Dixon	13	Native	
Rhodophyta	*Polysiphoniaatlantica* Kapraun & J.N.Norris	1	Native	
Rhodophyta	*Pterocladiellacapillacea* (S.G.Gmelin) Santel. & Hommers.	31	Native	
Rhodophyta	*Rhodymeniaholmesii* Ardissone	8	Native	
Rhodophyta	*Schotteranicaeensis* (J.V.Lamouroux ex Duby) Guiry & Hollenberg	1	Uncertain	New record
Rhodophyta	*Sphaerococcuscoronopifolius* Stackhouse	3	Native	
Rhodophyta	*Spyridiafilamentosa* (Wulfen) Harvey	1	Native	
Rhodophyta	*Symphyocladiamarchantioides* (Harvey) Falkenberg	5	Introduced	
Rhodophyta	*Tenareatortuosa* (Esper) M.Lemoine	1	Native	

**Table 4.** Summary of the macroalgal flora of Faial Island, with information on the species origins and status.

Phyllum	Order	Family	Specimens Number	Total taxa	Total species	Native	Introduced	Uncertain	Macaronesian endemism	New record
Rhodophyta	12	31	778	61	47	38	2	7	0	12
Chlorophyta	4	8	352	26	21	15	2	3	1	3
Ochrophyta	7	10	1117	30	24	21	1	2	0	8
Cyanobacteria	1	1	36	1	0	0	0	0	0	0
Total	24	50	2283	118	92	74	5	12	1	23
